# On meeting capital requirements with a chance-constrained optimization model

**DOI:** 10.1186/s40064-016-2110-z

**Published:** 2016-04-22

**Authors:** Ebenezer Fiifi Emire Atta Mills, Bo Yu, Lanlan Gu

**Affiliations:** School of Mathematical Sciences, Dalian University of Technology, Dalian, 116024 China

**Keywords:** Capital to risk asset ratio, Basel accord, CreditMetrics, Chance constraint

## Abstract

This paper deals with a capital to risk asset ratio chance-constrained optimization model in the presence of loans, treasury bill, fixed assets and non-interest earning assets. To model the dynamics of loans, we introduce a modified CreditMetrics approach. This leads to development of a deterministic convex counterpart of capital to risk asset ratio chance constraint. We pursue the scope of analyzing our model under the worst-case scenario i.e. loan default. The theoretical model is analyzed by applying numerical procedures, in order to administer valuable insights from a financial outlook. Our results suggest that, our capital to risk asset ratio chance-constrained optimization model guarantees banks of meeting capital requirements of Basel III with a likelihood of 95 % irrespective of changes in future market value of assets.

## Background

The financial crisis of 2008 reaffirmed the need to monitor risk in the banking sector as it has a ripple effect on the overall economy. The insolvency of most financial outfits at that time was as a result of increasing amounts of capital invested into long term and risky assets especially in the real estate market (Cannata and Quagliariello 2009; Brusov et al. 2015). Now more than ever, matters of subprime lending and the relevance of maintaining adequate capital to absorb adverse market conditions have become very important to bank managers and financial regulators (Erkens et al. 2012; Flannery 2014). Policy makers have recommended changes to already existing coordinated regulation to avert the probability of such occurrence in future. These changes include, significant increment in Capital to Risk (Weighted) Assets Ratio (CRAR) which is dependent on both the capital required and the capital resources to meet the requirements (Hasan et al. 2015). As a step in that direction, this paper’s primary aim is to use an asset-liability modeling approach via optimization techniques based on the CRAR to guarantee banks of meeting the stipulated regulation of Basel III with great likelihood irrespective of changes in forward market value of assets.

Asset-liability management in the banking sector primarily seeks to maximize profit through high returns on loans and other financial instruments, minimize risk as a result of mismatches between assets and liabilities and provide for liquidity needs (Choudhry 2012). Most banks manage their assets by issuing loans to creditors who have the capability to pay high interest rates and have minimal probability of default on their loans. Banks also diversify their investment portfolio and invest in securities with high returns and minimal risk in a bid to manage their assets. Over the years, several techniques and tools have been developed to aid bank managers in making the right investment decisions whilst reducing risk (see Szegö 2014; Bessis 2015; McNeil et al. 2015, for detailed reference). Policy makers have designed an internationally coordinated regulation known as the Basel Accord to ensure banks can absorb a reasonable and sufficient level of losses, which might otherwise threaten their solvency (Arnold et al. 2012; Lee 2014). Basel III bolsters and re-evaluates the global capital framework by increasing banks capital to risk (weighted) assets ratio (CRAR) requiring banks to raise capital defenses in times when credit is at excessive levels while upholding a financially sound banking environment which is the foundation of a functional market economy (Leignick and Wohltmann 2015). Banks manage the liquidity of their assets in order to meet reserve requirements such as the Third Basel Accord without incurring high costs.

Berger et al. (2008), Flannery and Rangan (2008) and Gropp and Heider (2010) show that banks CRAR respond to the shocks in the regulatory requirements and that banks actively manage their target capital levels that tend to exceed the minimally required ones. Athanasoglou et al. (2008) iterates the fact that banks with strong financial foundation and position pursue investment opportunities more effectively and have more time and awareness to counter problems such as unexpected losses thus attaining increased profitability. The application of the minimum capital to risk (weighted) assets ratio protects depositors and promotes the stability and efficiency of the financial systems. In their panel regressions on individual bank capital requirements in the UK, Bridges et al. (2014) observed that changes in micro prudential regulatory capital requirements affects capital ratios held by the banks and also affect lending with heterogeneous feedbacks in diverse sectors of the economy. Their finding shows how meeting capital a requirement is an important component of banks asset structure.

In this paper, we propose a chance-constrained optimization model by considering loan distribution follows a right truncated Gaussian distribution, to guarantee banks of coping with Basel III capital requirements even under the worst case scenario. We deal with an optimal portfolio model assuming that loans and a treasury bill are the investments made by the bank. The problem is faced under a theoretical perspective as well as simulation analysis. The introduction of a CRAR constraint characterized by the assumption made on the loan distribution, the construction of the deterministic convex counterpart of the CRAR chance constraint to obtain a tractable linear second order cone constraint and modified CreditMetrics approach represents the main novelty of this research paper.

To achieve our aim, we consider an optimization problem with the chance constraint of capital to risk (weighted) assets ratio under the condition that information regarding the distribution, mean and covariance of the risky asset in this case loan are known. Charnes and Cooper (1996) introduced chance-constrained programs. Over the years, significant literature have been established in this regard (see Prékopa 1995; Uryasev 2000; Delage and Mannor 2010; Barrera et al. 2014, for detailed discussion and applications). In finance and engineering, several problems can and have been constructed as chance-constrained stochastic linear programs of the form

1 $$\begin{aligned} \begin{aligned} \underset{x \in \mathbb {R}^{n}}{\text {minimize}}&\quad c^Tx \\ \text {subject to}&\quad \mathbb {P}\left\{ a_i(\zeta )^T x+b_i(\zeta ) \le 0, \; \forall i = 1, \ldots , m\right\} \ge 1-\epsilon _i\\& x\in \chi \end{aligned} \end{aligned}$$

where $x \in \mathbb {R}^n$ is the decision vector(investment allocations) and the uncertain constraint coefficients $a_i(\zeta ) \in \mathbb {R}^n$ and $b_i(\zeta ) \in \mathbb {R},\forall i = 1, \ldots , m$, depend affinely on random vector $\zeta \in \mathbb {R}^{h+1}$ whose distribution is assumed to be known. $1-\epsilon$, $\epsilon \in (0,1)$ is the risk level or safety factor usually given by the modeler. We thus have,

$$\begin{aligned} \begin{aligned} a_i(\zeta )&=a_i^0+\sum _{j=1}^{k}a_i^j \zeta _j\\ b_i(\zeta )&=b_i^0+\sum _{j=1}^{k}b_i^j \zeta _j \end{aligned} \end{aligned}$$

Auxiliary functions $y_i^j:\mathbb {R}^n \rightarrow \mathbb {R}$ are introduced for relaxation of notations and are defined through

$$\begin{aligned} y_i^j (x)=(a_i^j)^T x+b_i^j, \quad i=1,2,\ldots ,n, \quad j=0,1,\ldots ,k \end{aligned}$$

Therefore (1) can be rewritten as

2 $$\begin{aligned} \begin{aligned}&\mathbb {P}({a_i^0}^T x+b_i^0+\sum _{j=1}^{k}{a_i^j }^T\zeta _j+b_i^j \zeta _j)\le 0\\&\mathbb {P}(y_i^0(x)+\sum _{j=1}^{k} y^j_i(x)\zeta _j)\le 0 \end{aligned} \end{aligned}$$

For tractability purposes, we redefine the chance constraint (1) and rewrite (2) as a single generic constraint (Zymler et al. 2013), i.e. $i=1$ and $k=1$

3 $$\begin{aligned} \mathbb {P}\left\{ y^0(x)+y(x)^T \zeta \le 0 \right\} \ge 1-\epsilon \end{aligned}$$

By doing this, we convert the two uncertain parameters, $a_i(\zeta )\in \mathbb {R}^n$ and $b_i(\zeta )\in \mathbb {R}$ in (1) to one uncertain parameter, $\zeta \in \mathbb {R}^k$ in (2). We define the random vector $r = \left[1\quad \zeta ^T\right]^T\in \mathbb {R}^{h+1}$ where the first coordinate is a constant random variable and $\zeta$ is defined as forward market loan value which is estimated by modified CreditMetrics approach under a right truncated Gaussian distribution.

We consider all possible credit migration paths as opposed to sample credit migration paths in the CreditMetrics technical document by Gupton et al. (1997) and also make some other modifications. (see “CreditMetrics approach” section and “Appendix”). Luedtke (2014) and Yang and Xu (2016) pointed out that computation of the optimal solution of a chance-constrained program is notably hard to solve. Indeed, the only notable tractable case of the chance constraint formulation is by Calafiore and El Ghaoui’s (2006) paper when the chance constraint function is bilinear and the distribution of the uncertain data follows a set of radial probability distributions. Empirical evidence have revealed that the normal distribution is not appropriate to estimate credit risk (Crouhy et al. 2010). Returns in the credit markets are heavily skewed to the downside and therefore are non-normal (Gupton et al. 1997). Saunders and Allen (2010) observed that loans have acutely truncated upside returns and long downside risks and that the actual loan portfolio value distribution has negative skewness or a long-tailed downside. In this paper, we make an informed decision on the random vector *r* based on Saunders and Allen’s (2010) view on the actual loan portfolio distribution and assume that loan distribution can be described by truncated Gaussian distribution. One possible extension would be to consider, instead of truncated Gaussian distributed returns, the more general alpha-stable distributed returns (see (Rachev 2003; Rachev et al. 2005)).

The paper is organized as follows. Next section evaluates the probability constraint for right truncated Gaussian probability distribution and construct the deterministic convex counterpart of the chance constraint. The next section presents the CreditMetrics approach to estimate the forward market value of loans. The formulation of the optimization model based on the chance constraint of CRAR is structured and presented. We conduct a numerical example and illustrate the proposed method by a hypothetical example. Results and Concluding remarks are presented in the last section. Details and pseudocodes of the numerical procedure described in “CreditMetrics approach” section are given in “Appendix”.

## Chance constraint under truncated Gaussian distribution

Inspired by Calafiore (2006), we discuss a facet of the chance-constrained stochastic program (1). We evaluate the probability constraint for right truncated Gaussian probability distribution and construct specifically the deterministic convex counterpart of the chance constraint.

### Notation and parameter description

Without loss of generality, based on a standing assumption that the random constraints in (2) are independent, we make use of the single generic constraint

4 $$\begin{aligned} \mathbb {P}\left\{ y^0(x)+y(x)^T \zeta \le 0 \right\} \ge 1-\epsilon ,\;\epsilon \in (0,1) \end{aligned}$$

and define the random vector

$$\begin{aligned} r=\left[1 \quad \zeta ^T \right]^T \in \mathbb {R}^{h+1} \end{aligned}$$

and

$$\begin{aligned} \begin{aligned} {\hat{r}}&=E\{r\}=E\left\{ \left[1 \quad \zeta ^T\right]^T\right\}=\left[1 \quad {\hat{\zeta }}^T\right]^T\\ \Gamma&=var\{r\}=var\left\{ \left[1 \quad \zeta ^T\right]\right\} \end{aligned} \end{aligned}$$

Consider $v \le h+1$ as the rank of $\Gamma$ and $\Gamma _{f.r}\in \mathbb {R}^{h+1}$ as a full rank factor such that $\Gamma =\Gamma _{f.r}\Gamma _{f.r}^T$. Let

$$\begin{aligned} \tilde{z}=\left[y^0(x)\quad y(x)^T\right]^T\in \mathbb {R}^{h+1} \end{aligned}$$

We define the quantity

$$\begin{aligned} \varphi (z)= r^T \tilde{z} \end{aligned}$$

and

5 $$\begin{aligned} {\hat{\varphi }}(z)= E\{\varphi (z)\} \end{aligned}$$

6 $$\begin{aligned} \sigma ^2(z)= var\{\varphi (z)\}=\tilde{z}^T \Gamma \tilde{z} \end{aligned}$$

The normalized random variable is defined as

$$\begin{aligned} {\hat{\varphi }}(z)=\frac{\varphi (z)-{\hat{\varphi }}(z)}{\sigma (z)} \end{aligned}$$

Therefore, we can rewrite constraint (3) as

$$\begin{aligned} \mathbb {P}\{\varphi (z) \le 0\}=\mathbb {P}\{\tilde{\varphi }(z)\le -{\hat{\varphi }}(z)/\sigma (z)\}\ge 1-\epsilon \end{aligned}$$

### Formulation

#### **Definition 1**

A random vector $r\in \mathbb {R}^{h+1}$ is *Y*-radial with defining function $g(\cdot )$ if $r-E(r)=Y\eta$ where $Y \in \mathbb {R}^{h+1,v}$ is a full rank matrix of a fixed nature and $\eta \in \mathbb {R}^{v}$ is a random vector with probability density function $f_\eta$ that is dependent exclusively on the norm of $\eta$ i.e. $f_\eta (\eta )=g(\Vert \eta \Vert )$.

From the above definition, the covariance of $\eta$ is

$$\begin{aligned} \Sigma _{\eta }=\left( V_v \int _{0}^ \infty w^{v+1}g(w)dw \right) I_v \end{aligned}$$

where $V_v$ is the Euclidean volume of the standard unit ball of radius $\mathbb {R}^v$. The variance of *r* is defined as

$$\begin{aligned} var\{r\}=\left( V_v \int _{0}^ \infty w^{v+1}g(w)dw \right) QQ^T \end{aligned}$$

Given that $var\{r\}=\Gamma$, then $Y=s \Gamma _{f.r},\quad s=\left( V_v \int _{0}^ \infty w^{v+1}g(w)dw \right) ^{-1/2}$ where $\Gamma _{f.r}$ is a full rank factor of $\Gamma$ i.e. $\Gamma =\Gamma _{f.r}\Gamma _{f.r}^T$. The possibly singular multivariate *b* right truncated Gaussian distribution in $\mathbb {R}^{h+1}$, with mean $\tilde{r}$ and covariance $\Gamma$ is *Y*-radial with $Y=s\Gamma _{f.r}$ and defining function

$$\begin{aligned} g(w)=\frac{1}{\left\{ \sigma \Phi \left( \frac{b-\mu }{\sigma }\right) \right\} ^s}\phi \left( \frac{w-\mu }{\sigma }\right) \end{aligned}$$

If *r* is *Y*-radial and has defining function *g*(*w*) and covariance $\Gamma$, then

$$\begin{aligned} \begin{aligned} \tilde{\varphi }(z)&=\tilde{z}Y\eta /\sigma (z)=s \tilde{z}^T \Gamma _{f.r} \eta /\sqrt{\tilde{z}^T \tilde{z}}\\ \mathbb {P}\{\varphi (z) \le 0\}&=\mathbb {P}\{\tilde{\varphi }(z)/s \le -{\hat{\varphi }}(z)/s\sigma (z)\}=\mathbf {F}_{\tilde{\varphi }(z)/s}(-{\hat{\varphi }}(z)/s\sigma (z)) \end{aligned} \end{aligned}$$

and the cumulative density function is defined as

$$\begin{aligned} \mathbf {F}_{\tilde{\varphi }(z)/s}(-{\hat{\varphi }}(z)/s\sigma (z))=\int _{-\infty }^{-{\hat{\varphi }}(z)/s\sigma (z)} f_{\tilde{\varphi }(z)/s}(L)dL \end{aligned}$$

Therefore, $\mathbb {P}\{\varphi (z) \le 0\}\ge 1-\epsilon$ is equivalent to $s\mathbf {F}^{-1}(1-\epsilon )\sigma (z)+{\hat{\varphi }}(z)\le 0$ for $\epsilon \in (0,1)$.

A *b* right truncated Gaussian distribution with covariance $\Gamma$ and mean ${\hat{r}}$ is *Y*-radial with $Y=\Gamma _{f.r},\,s=1$ and defining function

$$\begin{aligned} g(w)=\frac{1}{\sigma \Phi \left( \frac{b-\mu }{\sigma }\right) }\phi \left( \frac{w-\mu }{\sigma }\right) \end{aligned}$$

Appropriately, $f_{\tilde{\varphi }(z)/s}$ is the *b* right truncated Gaussian density function

$$\begin{aligned} f_{\tilde{\varphi }(z)/s}(L)=\frac{1}{\sigma \Phi \left( \frac{b-\mu }{\sigma }\right) }\phi \left( \frac{L-\mu }{\sigma }\right) \end{aligned}$$

and $\mathbf {F}$ is the standard *b* right truncated Gaussian distribution cumulative probability function

$$\begin{aligned} \mathbf {F}(L)=\mathbf {F}_{RG}(L)=\int _{-\infty }^L \frac{1}{\Phi ({b})}\phi (t)dt=\frac{1}{\Phi ({b})}\Phi (L) \end{aligned}$$

and the inverse for the standard *b* right truncated Gaussian distribution cumulative probability function is

$$\begin{aligned} \mathbf {F}^{-1}(L)=\mathbf {F}^{-1}_{RG}(L)=\Phi ^{-1}(\Phi (b)\cdot L) \end{aligned}$$

#### **Theorem 1**

*The chance constraint,*$\mathbb {P}\{{\hat{r}}^T \tilde{z} \le 0 \}\ge 1-\epsilon$, *for any*$\epsilon \in (0,1)$*whererhas abright truncated Gaussian distribution with defining function g(w), covariance*$\Gamma$*and* mean ${\hat{r}}$*with*$s=1$*is equivalent to the second order cone (SOC) constraint*$\mathbf {F}^{-1}_{RG}(1-\epsilon )\sqrt{\tilde{z}\Gamma \tilde{z}}+{\hat{r}}^T\tilde{z}\le 0$.

## CreditMetrics approach

Future bank capital is dependent on the forward market values of assets as well as liabilities. We use a modified CreditMetrics approach to estimate the forward market value of loans which is the uncertainty parameter in (3). CreditMetrics from JP Morgan, made public and published in 1997, is reviewed in this section. The methodology estimates the forward distribution of the changes in portfolio value of loan products at a specified time horizon, typically one year. The changes in value are associated with the migration in credit quality of the obligor, both up and downgrades, including default. We develop a Monte Carlo framework from a modified CreditMetrics approach (see “Appendix”) to include all possible migrations and scenarios. This method is adopted for the purpose of calculating the forward market value of loans and its associated risk measure.

### Rating migration

Based on historical data on loan, Standard and Poor (S&P) construct a transition matrix which shows the probability of a rated loan being downgraded, upgraded or defaulting during a given period, usually one year. The estimated probabilities of average one-year corporate transition rates (1981–2013) of corporations based in Europe are shown in Table 1. Each cell in the transition matrix has a probability. The rating sections in the rows of the transition matrix signify current credit ratings and the columns of the transition matrix show expected credit rating at the end of the year. For example, the $P(BBB \rightarrow BB) = 0.0431$ show the chances of a *BBB*-rated loan downgrading to B over the ensuing year.

Recovery rates and the probability of default are essential parameters in credit risk modeling. Calabrese (2008) defines recovery rate as the payback quota of the loan. Considerable studies on recovery rates on corporate bonds have been performed (Altman 1989; Schuermann 2004; Renault and Scaillet 2004; Jankowitsch et al. 2014) and Asarnow and Edwards (1995), Calabrese and Zenga (2008), Khieu et al. (2012) and Calabrese (2008) have all investigated bank loans. Carty and Lieberman (1996) and Schuermann (2004) made an interesting revelation by showing that the mean of bonds is lower than the average recovery rate of bank loans. Calabrese and Zenga (2008) measured the recovery rates on non-performing bank loans in Italy by using the boundary property [0, 1] in computing the loan recovery rate as the ratio between capitalized actual recovery amount and capitalized total exposure, where the latter parameter is the sum of Exposure-At-Default , capitalized legal costs and interest on delayed payment. We employ loan recovery rates estimated in Calabrese and Zenga (2008) for Italy under categories of secured and unsecured loans shown in Table 2 for the estimation of forward value of loans via a modified CreditMetrics approach.

**Table 1:** Average 1-year corporate transition rates for Europe (1981–2013) (%). *Source:* Standard & Poor’s Annual Global Corporate Default Study & Rating Transitions (March 2014)

RATING	AAA	AA	A	BBB	BB	B	CCC/C	D	Not rated
AAA	83.12	10.76	0.63	0.21	0.00	0.00	0.21	0.00	5.06
AA	0.28	84.32	10.92	0.66	0.00	0.00	0.00	0.00	3.81
A	0.02	2.17	85.95	6.36	0.24	0.02	0.00	0.05	5.20
BBB	0.00	0.12	4.26	82.99	4.31	0.50	0.12	0.10	7.70
BB	0.00	0.00	0.13	4.92	71.46	8.02	0.57	0.57	14.33
B	0.00	0.00	0.07	0.49	7.52	68.31	4.85	3.30	15.46
CCC/C	0.00	0.00	0.00	0.00	0.00	16.22	31.76	31.76	20.27

**Table 2:** Loan recovery rates of Italy by secured status $(\%)$. *Source:* Calabrese and Zenga (2008)

Status	Secured	Unsecured
Mean	56.66	37.98
Standard deviation	30.44	33.87

**Table 3:** One-year forward zero curve for each credit rating category (%). *Source:* CreditMetrics, JP Morgan

Category	Year 1$(f_{cr}^{1,2})$	Year 2 $(f_{cr}^{1,3})$	Year 3 $(f_{cr}^{1,4})$	Year 4 $(f_{cr}^{1,5})$
AAA	3.60	4.17	4.73	5.12
AA	3.65	4.22	4.78	5.17
A	3.72	4.32	4.93	5.32
BBB	4.10	4.67	5.25	5.63
BB	5.55	6.02	6.78	7.27
B	6.05	7.02	8.03	8.52
CCC/C	15.05	15.02	14.03	13.52

### Valuation

In this section, we calculate forward value of loans at the end of the risk horizon, 1 year. However, since there are 8 possible credit ratings in the transition matrix from Table 1, we have to estimate values based on all possible migration paths given maturity dates. In the worst case scenario of the loan i.e. default, two input variables are required the secured status of the loan and its associated recovery rate.

Valuation is done based on all the possible migration paths, residual cash flows and from the one-year forward zero-curve associated with the rating of the issuer. Expected upgrade or downgrade in credit rating leads to changes in the value of the loan. The magnitude of these changes are accessible with the estimation of forward zero curves for each rating Abaffy et al. (2007).

**Table 4:** Possible migration paths of a 2 year BBB-rated loan

	Non-default m = 0	Migration m = 1	Paths m = 2		Default m = 0	Migration m = 1	Paths m = 2
1	BBB	AAA	AAA	1	BBB	D	
2	BBB	AAA	AA	2	BBB	AAA	D
3	BBB	AAA	A	3	BBB	AA	D
4	BBB	AAA	BBB	4	BBB	A	D
5	BBB	AAA	BB	5	BBB	BBB	D
6	BBB	AAA	B	6	BBB	BB	D
7	BBB	AAA	C	7	BBB	B	D
8	BBB	AA	AAA	8	BBB	C	D
9	BBB	AA	AA				
10	BBB	AA	A				
11	BBB	AA	BBB				
12	BBB	AA	BB				
13	BBB	AA	B				
14	BBB	AA	C				
15	BBB	A	AAA				
16	BBB	A	AA				
17	BBB	A	A				
18	BBB	A	BBB				
19	BBB	A	BB				
20	BBB	A	B				
21	BBB	A	C				
22	BBB	BBB	AAA				
23	BBB	BBB	AA				
24	BBB	BBB	A				
25	BBB	BBB	BBB				
26	BBB	BBB	BB				
27	BBB	BBB	B				
28	BBB	BBB	C				
29	BBB	BB	AAA				
30	BBB	BB	AA				
31	BBB	BB	A				
32	BBB	BB	BBB				
33	BBB	BB	BB				
34	BBB	BB	B				
35	BBB	BB	C				
36	BBB	B	AAA				
37	BBB	B	AA				
38	BBB	B	A				
39	BBB	B	BBB				
40	BBB	B	BB				
41	BBB	B	B				
42	BBB	B	C				
43	BBB	C	AAA				
44	BBB	C	AA				
45	BBB	C	A				
46	BBB	C	BBB				
47	BBB	C	BB				
48	BBB	C	B				
49	BBB	C	C				

From Table 3, $f_{cr}^{1,j}$ is the $(j-1)$-year forward rate with credit rating *cr* thus for year 1, we have $f_{cr}^{1,2}$ and so on. By this interpretation, the entries of Table 3 are spot rates from now. Consider $\tilde{f}_{cr}^{i,i+1}$ as the one-year forward rate with its associated credit rating $cr_i$ from the end of the *i*-th year to the end of the $(i+1)$-th year. Therefore, the one-year forward rate can be estimated by

7 $$\begin{aligned} \begin{aligned}&\tilde{f}_{cr_1}^{1,2}={f}_{cr_1}^{1,2}, \quad i=1\\&\quad (1+{f}_{cr_i}^{i,i+1})^i=(1+\tilde{f}_{cr_i}^{i,i+1})(1+{f}_{cr_i}^{1,i})^{i-1}, \quad i=2,\ldots ,m-1 \end{aligned} \end{aligned}$$

The discount factor of the cash flow from the end of the *j*th year to the end of the first year for loan type *k* can be computed by the following

8 $$\begin{aligned} \begin{aligned}&d_{k1}^{-1}=1,\quad j=1\\&d_{kj}^{-1}=\prod _{i=1}^{j-1}\left( 1+\tilde{f}_{cr_i}^{i,i+1}\right) =\prod _{i=1}^{j-1}\frac{(1+{f}_{cr_i}^{i,i+1})^i}{(1+{f}_{cr_i}^{1,i})^{i-1}},\quad j=2,\ldots ,m \end{aligned} \end{aligned}$$

The forward loan value is the sum of discounted cash flows of each year corresponding to possible credit rating transition paths till maturity *m*. Then the forward value, $\zeta _{kt}$ of a unit capital invested in the *k*th loan with a non-default credit rating migration path *t* is

9 $$\begin{aligned} \zeta _{kt}=\sum _{j=1}^{m-1}R_k d_{kj}+(1+R_k) d_{km} \end{aligned}$$

where $R_k$ is the interest rate of the *k*th loan.

In case *k*th loan defaults before or at the end of the period $q=1,2,3,\ldots ,m$, then the forward value, $\zeta _{kdt}$, of a unit capital invested in the *k*th loan with a default credit rating migration path *dt* is

10 $$\begin{aligned} \zeta _{kdt}=\sum _{j=1}^{q-1}R_k d_{kj}+RR_k d_{km} \end{aligned}$$

where $RR_k$ is the recovery rate if the *k*th loan which is dependent on the secured status in Table 2.

Table 4 shows the possible migration paths for a 2 year BBB-rated loan. There are 49 non-default paths and 8 default paths. In general, the *k*th loan with maturity *m* has $7^m$ non-default possible credit rating migration paths. The *k*th loan with maturity *m* has $\sum _{q=1}^{m} 7^{q-1}$ default possible credit rating migration paths.

**Table 5:** Asset correlations among loans

Risky assets	$\zeta _1$	$\zeta _2$	$\zeta _3$	$\zeta _4$	$\zeta _5$
$\zeta _1$	1	0.15	0.1	0.1	0.1
$\zeta _2$	0.15	1	0.2	0.15	0.1
$\zeta _3$	0.1	0.2	1	0.2	0.1
$\zeta _4$	0.1	0.15	0.2	1	0.25
$\zeta _5$	0.1	0.1	0.1	0.25	1

### Expected value and credit risk

Gupton et al. (1997) and Benz (2002) observed that credit returns are heavily skewed to the downside and therefore are non-normal. Saunders and Allen (2010) also made an interesting revelation that loans have both acutely truncated upside returns and also long downside risks and that the actual loan portfolio value distribution exhibits a long-tailed downside or negative skewness. We therefore make an informed decision based on empirical evidence on the actual loan portfolio distribution and assume that the *k*th loan year-ahead or forward value, $\zeta _k$, distribution is described by the right truncated Gaussian distribution. The long downside tail of the distribution of loan returns is caused by defaults.

Consider the credit migration path satisfying the Markov property, then the migration probability of the *k*th loan with *t* non-default path is

11 $$\begin{aligned} P_{kt}=\prod _{i=0}^{m-1}P_{cr_{i},cr_{i+1}},\quad t=1,\ldots ,7^m \end{aligned}$$

and for default path

12 $$\begin{aligned} P_{kdt}=\prod _{i=0}^{q-1}P_{cr_{i},cr_{i+1}},\quad t=1,\ldots ,N \qquad N=\sum _{q=1}^m 7^{q-1} \end{aligned}$$

where $P_{cr_{i},cr_{i+1}}$ signify the probability of migration from $cr_i$ credit rating at the end of the *i*th year to $cr_{i+1}$ credit rating at the end of $(i+1)$-th year.

By the assumption of the forward loan value of unit capital invested in the *k*th loan following a right truncated Gaussian distribution at *b*, then the expected forward loan (mean) value and variance of unit capital invested in the *k*th loan are

13 $$\begin{aligned} E(\zeta _k\mid \zeta _k \le b)=\mu _k-\sigma _k \left[ \frac{\phi (\beta )}{\Phi (\beta )}\right] ,\quad \mu _k=\sum _{t=1}^{7^m} \zeta _{kt} P_{kt}+\sum _{t=1}^{N} \zeta _{kdt} P_{kdt} \end{aligned}$$

and

14 $$\begin{aligned}&Var(\zeta _k \mid \zeta _k \le b)= \sigma ^2_k \left[ 1-\beta \frac{\phi (\beta )}{\Phi (\beta )}-\left( \frac{\phi (\beta )}{\Phi (\beta )}\right) ^2\right] , \quad \sigma ^2_k=\sum _{t=1}^{7^m}(\zeta _{kt}-\mu _k)^2 P_{kt}\nonumber \\ &\quad +\sum _{t=1}^{N}(\zeta _{kdt}-\mu _k)^2 P_{kdt} \end{aligned}$$

respectively.

## Formulation of the model

The optimization model based on the chance constraint of capital to risk (weighted) asset requirement is structured and presented in this section. We first determine the objective function and then proceed to construct its related constraints.

### Objective function

Let $R=[R_1,R_2,\ldots ,R_u,R_{u+1},\dots ,R_{u+v}]^T$ be the vector of annual interest rate of loans and treasury bill, fixed assets and non-interest earning assets (riskless) with *u* corresponding to the loans and *v* as the riskless assets. Denote $x=[x_1,x_2,\ldots ,x_u,x_{u+1},\ldots ,x_{u+v}]^T$ as the vector of asset allocation or investment proportion which is the decision variable. Then the objective function can be defined as

15 $$\begin{aligned} \begin{aligned}&\underset{x}{\text {max}}&R^Tx \end{aligned} \end{aligned}$$

### Constraints

#### CRAR constraint

Denote Q as the bank’s total asset amount and *TL* be the bank’s total liability. Let $\Omega =[\zeta ^T,\xi ^T]^T$ be a unit vector of assets with $\zeta =[\zeta _1,\zeta _2,\ldots ,\zeta _u]^T$ and $\xi =[\xi _1,\xi _2,\ldots ,\xi _v]^T$ corresponding to loans and riskless assets (treasury bill, fixed assets and non-interest earning assets) respectively. $\zeta$ constitutes uncertain parameters which can be estimated (see “Valuation” section) and $\xi$ is a deterministic vector of $[1+R_{u+1},1+R_{u+2},\ldots ,1+R_{u+v}]^T$. Let $\varpi _k$ denote the *k*th asset’s weight factor and $1-\epsilon$ is the safety factor.

According to Basel III, the minimum total capital requirement is 8 %. Basel III introduced two additional capital buffers; capital conservation buffer and countercyclical buffer. Capital conservation buffer of 2.5 % is designed to ensure that banks build up capital buffers outside periods of stress which can be drawn down as losses are incurred. The countercyclical buffer range of 0 to 2.5 % which is set by national regulators aims to ensure that banking sector capital requirements take account of the macro-financial environment in which banks operate.

Based on the definition of CRAR i.e.

$$\begin{aligned} {\textit{CRAR}} = \frac{Q\Omega ^Tx-TL}{Q(\varpi _1\zeta _1x_1+\ldots +\varpi _u \zeta _ux_u+\varpi _{u+1}\xi _{u+1}x_{u+1}+\ldots +\varpi _{u+v}\xi _{u+v}x_{u+v})}\ge \lambda \end{aligned}$$

the following holds

16 $$\begin{aligned} \mathbb {P}\left( \frac{Q\Omega ^Tx-TL}{Q(\varpi _1\zeta _1x_1+\ldots +\varpi _u\zeta _ux_u+\varpi _{u+1}\xi _{u+1}x_{u+1}+\ldots +\varpi _{u+v}\xi _{u+v}x_{u+v})}\ge \lambda \right) \ge 1-\epsilon \end{aligned}$$

As the moment information (mean and variance) of the forward or year-ahead value of $\zeta$ corresponds to all the possible migration paths of loans credit ratings, constraint (16) means even with the changes in the risky assets credit ratings, the bank can still meet the total requirements of the third Basel Accord with greater likelihood. The chance-constrained model based on capital adequacy ratio (16) can be written in terms of a single generic constraint as

17 $$\begin{aligned} \mathbb {P}\left\{ y^0(x)+y(x)^T\zeta \le 0\right\} \ge 1-\epsilon \end{aligned}$$

where

$$\begin{aligned} \begin{aligned}&y^0(x)=TL-Q\sum _{k=u+1}^{u+v}(1+R_k)x_k\\&y(x)=Q\sum _{k=1}^{u}(\lambda \varpi _k-1)x_k \end{aligned} \end{aligned}$$

##### *Proof*

From the definition of CRAR i.e.

$$\begin{aligned} \frac{Q\Omega ^Tx-TL}{Q(\varpi _1\zeta _1x_1+\ldots +\varpi _u\zeta _ux_u+\varpi _{u+1}\xi _{u+1}x_{u+1}+\ldots +\varpi _{u+v}\xi _{u+v}x_{u+v})}\ge \lambda \end{aligned}$$

where the riskless asset weight factor is zero i.e.$\sum _{k=u+1}^{u+v}\varpi _k=0$, therefore the following holds

$$\begin{aligned} \begin{aligned}&Q\Omega ^Tx-TL\ge \lambda Q \left( \sum _{k=1}^{u} \varpi _k \zeta _k x_k \right) \\&Q\sum _{k=1}^{u}\zeta _kx_k+Q\sum _{k=u+1}^{u+v}x_k+Q\sum _{k=u+1}^{u+v} R_kx_k-TL-\lambda Q \left( \sum _{k=1}^{u}\varpi _k \zeta _k x_k \right) \ge 0\\&-Q\sum _{k=1}^{u}\zeta _kx_k-Q\sum _{k=u+1}^{u+v}x_k-Q\sum _{k=u+1}^{u+v} R_kx_k+TL+\lambda Q \left( \sum _{k=1}^{u}\varpi _k \zeta _k x_k \right) \le 0\\&TL+Q \sum _{k=1}^{u}(\lambda \varpi _k-1)\zeta _k x_k-Q \sum _{k=u+1}^{u+v}(1+R_k)x_k \le 0\\&TL-Q \sum _{k=u+1}^{u+v}(1+R_k)x_k +Q \sum _{k=1}^{u}(\lambda \varpi _k-1)\zeta _k x_k\le 0,\\ \end{aligned} \end{aligned}$$

$$\square$$

which was set out to proof.

##### **Proposition 1**

*From the mathematical formulation in the * “Notation and parameter description” *section, under the assumption of known distribution ofras truncated Gaussian distribution, for any*$\epsilon \in (0,1),$*constraint* (17) *is equivalent to*$\mathbf {F}_{RG}^{-1}(1-\epsilon )\sigma (\varphi (z))+{\hat{\varphi }}(z)\le 0$.

#### Constraint based on other factors

Other constraints to control banks liquidity risk as well as other constraints coping with the defined goals of the bank are constructed here. We assume the set of admissible assets is a nonempty polyhedral set and denote it as $\mathcal {X}=\{x:Bx=f ,Gx=h\}$ where *B* is a $m \times n$ matrix, *f* is a *m*-dimensional vector, *G* is a $p \times n$ matrix and *h* is a *p*-dimensional vector. In particular, one of the constraints in the set $\mathcal {X}$ is

18 $$\begin{aligned} \sum _{k=1}^{u+v} x_k=1 \end{aligned}$$

which satisfies the requirements of the sum of all investment allocations to be 1. Other constraints can be constructed based on the requirement of setting up limit allocation ratio of the investment.

### The optimization model

The asset-liability model via optimization techniques based on the capital asset to risk (weighted) ratio (CRAR) to guarantee banks of coping with the stipulated regulation of Basel III with great likelihood irrespective of changes in forward market value of assets is defined as

19 $$\begin{aligned} \begin{aligned} \underset{x \in \mathbb {R}^{n}}{\text {maximize}}&\quad R^Tx \\ \text {subject to}&\quad \mathbf {F}_{RG}^{-1}(1-\epsilon )\sigma (\varphi (z))+{\hat{\varphi }}(z)\le 0\\&\sum _{k=1}^{u+v} x_k=1\\&x_1,x_2,\ldots ,x_{u+v} \ge 0\\ \end{aligned} \end{aligned}$$

Therefore, the CRAR chance-constrained asset-liabilty optimization model based on the informed assumption and decision that the uncertain parameter , $\zeta$ (forward value of a loan) can be described by a *b* right truncated Gaussian distribution transforms to a tractable second-order cone program (SOCP), which has a solution in polynomial time, refer to Alizadeh and Goldfarb (2003) and Maggioni et al. (2009).

**Table 6:** Asset Structure of an Italian Bank

Assets	Collateral status	Interest rate $(\%)$	Recovery rate $(\%)$	Risk weights $(\%)$	Mean	Variance	Allocation
$(\zeta _1)$ 3 year AAA commercial and industrial loan	Inventory or account receivables	4.98	56.66	20	0.9143	0.0196	0.0010
$(\zeta _2)$ 5 year AA agriculture and farm loan	Land, equipment, crops, livestock etc	5.71	56.66	50	0.8696	0.0347	0.1664
$(\zeta _3)$ 2 year BBB personal loan	Unsecured	6.51	37.98	75	0.9247	0.0233	0.1121
$(\zeta _4)$ 3 year B education loan	House, land etc	5.87	56.66	75	0.6215	0.0929	0.4192
$(\zeta _5)$ 4 year A vehicle loan	Savings account or car itself	5.14	56.66	75	0.8451	0.0360	0.2912
1 year treasury bill	Not applicable	0.8	100	0	1.008	0	0.0101
Fixed assets	Not applicable	0	100	0	1	0	
Non-interest earning assets	Not applicable	0	100	0	1	0	

## Numerical example

In this section we test the capability of CRAR chance-constrained asset-liability optimization model on a hypothetical bank operating from Italy. Loan information are somewhat private information. We therefore use datasets with an informed suppositional background. We define and back the data used for the experiment with references and trusted sources.

### Data description

From the asset structure of a bank operating from Italy, let *Q* the bank’s total asset amount be €1500000 and the bank’s total liability *TL* equal €1192000. The financial assets characterizing financial environment on the bank are 5 loan types, a treasury bill, fixed assets and non-interest earning assets. We treat treasury bill, fixed assets and non-interest earning assets as riskless assets and the 5 loan types as risky assets. Table 6 shows all necessary information about the financial assets including investment proportions to be allocated. The expected forward or year-ahead market value of loan types (13) and its variance (14) are estimated with consideration of all possible migration paths including default.

Interest rate (*R*) for individual loans are set to lending interest rates for Italy quoted by World Bank in 2013 as a reference point. The treasury bill rate of $0.8\%$ for Italy in 2013 was also provided by World Bank and risk weights are based on the standardized approach (credit risk) of Basel Accord. The recovery rates for loan types are set according to their collateral status shown in Table 2.

Suppose the correlation coefficent of loans is between $10\%$ and $30\%$, with the aid of Table 11.2 in Gupton et al.’s (1997) CreditMetrics technical document we consider the correlations among loan borrowers as shown Table 5.

**Table 7:** Expected value of year-ahead market value of loans under worst case scenario

Loan type	Worst-credit migration path	Value
3 Year AAA commercial and industrial loan	$AAA \rightarrow CCC \rightarrow CCC \rightarrow D$	0.5214
5 Year AA agriculture and farm loan	$AA\rightarrow CCC\rightarrow CCC \rightarrow CCC \rightarrow CCC \rightarrow D$	0.5296
2 Year BBB personal loan	$BBB \rightarrow D$	0.3798
3 Year B education loan	$B \rightarrow CCC \rightarrow CCC \rightarrow D$	0.5380
4 Year A vehicle loan	$AAA \rightarrow CCC \rightarrow CCC \rightarrow CCC\rightarrow D$	0.5171

### The CRAR chance constraint optimization model

In this section we present the CRAR chance constraint optimization model. In order to fully understand the model, mathematical computations are made in that regard to obtain a tractable linear SOCP problem.

#### Objective function

Equation (15) is filled with entries of interest rate column of Table 6. Thus,

20 $$\begin{aligned} \begin{aligned} \underset{x}{\text {maximize}}&\quad 0.0498x_1+0.0571x_2+0.0651x_3+0.0587x_4+0.0514x_5+0.0080x_6 \end{aligned} \end{aligned}$$

#### CRAR constraint

Bank management usually allot proportions of sum total asset amount, *Q*, to riskless assets; in this case fixed assets and non-interest earning assets, before making any investment. In this regard, we assume bank management allocate €480000 to fixed assets and €420000 to non-interest earning assets.Therefore, we denote total amount for investment in loans $(\zeta )$ and treasury bill $(\xi _1)$ as *A* = €600000. We employ capital to risk (weighted) assets ratio (CRAR) proposed by Basel III as the stipulated regulation requirement. The minimum total capital requirement is 8 %. Basel III introduced two additional capital buffers; capital conservation buffer of 2.5 % and countercyclical buffer of 0 to 2.5 %. For the purpose of this study, we assume a total capital requirement ratio, $\lambda$, of 11 % and a safety factor of 0.95. The constraint based on CRAR is

21 $$\begin{aligned}&\mathbb {P}\left( \frac{A(\zeta ^T x+\xi _1x_6)+900000-1192000}{A(0.2\zeta _1 x_1+0.5\zeta _2 x_2+\ldots +0.75 \zeta _5x_5)}\ge 0.11\right) \ge 0.95 \end{aligned}$$

22 $$\begin{aligned}&\mathbb {P}\left\{ y^0(x)+y(x)^T\zeta \le 0\right\} \ge 0.95 \end{aligned}$$

where

$$\begin{aligned} \begin{aligned} y^0(x)&=1192000-600000 \,(1+0.0080)x_6-480000-420000\\ y(x)^T&=600000( (0.11\times 0.2-1)x_1+(0.11\times 0.5-1)x_2+\ldots +(0.11\times 0.75-1)x_5 ) \end{aligned} \end{aligned}$$

Equation (21) is equivalent to

23 $$\begin{aligned} 1.4639\sqrt{\tilde{z}\Gamma \tilde{z}}+{\hat{r}}^T \tilde{z}\le 0 \end{aligned}$$

where

$$\begin{aligned} \tilde{z}= & {} \begin{bmatrix} 292000-604800x_6\\ -586800x_1\\ -567000x_2 \\ -550500x_3\\ -550500x_4\\ -550500x_5\\ \end{bmatrix}\\ \Gamma= & {} \begin{bmatrix} 0&0&0&0&0&0\\ 0&0.0196&0.0039&0.0021&0.0043&0.0027\\ 0&0.0039&0.0347&0.0057&0.0085&0.0035\\ 0&0.0021&0.0057&0.0232&0.0093&0.0029\\ 0&0.0043&0.0085&0.0093&0.0929&0.0145\\ 0&0.0027&0.0035&0.0029&0.0145&0.0360\\ \end{bmatrix} \end{aligned}$$

and

$$\begin{aligned} \hat{r}^T=[1 \quad 0.9143 \quad 0.8696 \quad 0.9247 \quad 0.6215 \quad 0.8451] \end{aligned}$$

#### Constraint based on other factors

With requirements of investment allocations summing up to one, the constraint below holds.

24 $$\begin{aligned} \sum _{k=1}^6 x_k=1 \end{aligned}$$

From an investment principle, we set up a constraint of nonnegativity with respect to loan allocations as

25 $$\begin{aligned} x_1,\ldots ,x_5 \ge 0 \end{aligned}$$

Assuming bank management places an allocation limit ratio of 0.01 on treasury bill as a result of its riskless property, then

26 $$\begin{aligned} x_6\ge 0.01 \end{aligned}$$

The chance-constrained optimization model is converted into a tractable linear SOCP by combining equations (20) and (23–26).

## Results and conclusion

The modified CreditMetrics method described earlier was coded in Matlab(R2015a). All computations were performed on a MacBook Pro (Intel(R) Core(TM) i7-3615QM CPU @ 2.30GHz, 16GB RAM).

Column 8 of Table 6 displays entries of the optimal weights of loans and treasury bill. The 3-year AAA Commercial and Industrial Loan allocation value of 0.0010 is the least amongst the optimal investment allocation values. This may be attributed to its smaller risk weight and interest rate. We observe that an increment in the total liability leads to higher allocation to the treasury bill and vice versa. This is due to the model guarding against loss at the expense of a higher interest. The optimal investment portfolio interest income is 0.0565 which is obtained by computations made by CVX package via Matlab (R2015a).

To further understand the results of the optimization model, we explore CRAR chance constraint with the utilization of optimal investment allocation ratios and worst credit migration path values of loans. The expected values of year-ahead market value of loans under worst case scenario are assigned to $\zeta$, the optimal allocation proportions to *x* and $\xi _1$ equals 1.008.

Using the loan values under the worst-credit migration path from Table 7, the CRAR value of 0.0860 is reported. We take this approach for the purpose of testing the model under worst-case scenario of default. The value of CRAR even under worst case scenario confirms the bank is guaranteed of coping with Basel III minimum total requirement of 0.08 with great likelihood of $95\,\%$.

The paper presented a chance-constraint optimization model that guarantees a bank, with treasury bills, fixed assets, non-interest earning assets and loans, to cope with capital requirements of Basel III with a probability of 95%, whichever the future value of assets will be. The findings of this research work explicitly disclose that the total proportion of CRAR’s lower than Basel III minimum capital requirement is less than the risk probability of 5 %. Therefore, the chance-constrained CRAR optimization model does guarantee banks of meeting capital requirements of Basel III with greater likelihood of 95 % irrespective of changes in forward market value of assets.

## Appendix

In this “Appendix” we provide details and pseudocodes of the numerical procedure described in “CreditMetrics approach” section.

The first step of our experiment is represented by the simulation of non-default and default paths. The general procedure for implementation are as follows:

*Step A* Simulation of all non-default and default paths for maturity period *m*.

In this step all non-default and default paths are generated for maturity $M=1,\ldots ,m$ years. The output for every *M* in a non-default path is a matrix of size $7^{m}\times M$ where rows correspond to a path and columns indicate credit rating at the end of the year. All the *m* default path matrices are stored in a cell array of length *m*. The output for every *M* in a default path is a matrix of size $7^{(m-1)}\times M$. But unlike non default paths, for default paths with maturity *m* the possible paths are all paths in all matrices from $M =1,\ldots ,m$. The indexing scheme of $AAA \rightarrow 1, AA \rightarrow 2, A \rightarrow 3, BBB \rightarrow 4, BB \rightarrow 5, B \rightarrow 6, CCC \rightarrow 7$ and $D \rightarrow 8$ is used.

The pseudocode is as follows:

- set the maturity period $M=1$;
- initialize a zero matrix of size $7^{M} \times M$;
- convert decimal number $W = 0$ to base 7 of length *M* and store it in the matrix row $R = 1$;
- set $$W= W+1$$ and $$R= R+1$$;
- if $W<7^m$, repeat steps 3a and 4a ;
- add 1 to all the elements in the matrix;
- store the resultant matrix in cell array $migN\{M\}$, the non-default migration path;
- store 8 in the $1\times 1$ default path matrix (direct default in first year);
- add it to cell array $migD\{M\}$, default migration path;
- get the matrix from $migN\{M-1\}$;
- append the matrix with a column of 8’s and add to $migD\{M\}$;
- set $$M=M+1$$;
- if $M<m$, repeat steps 2a to 13a;

*Step B* Calculating discount factors.

This step precomputes and stores discount factors for all transition probabilities for $m =1,\ldots ,5$. The procedure runs as follows:

1. load forward rates data from Table 3;
2. append a column of 1s in *dFactor* first column i.e. $dFactor\{1\}=ones(7,1)$;
3. for every $M=1,\ldots ,m$ and for every path for both *migD* and *migN*, calculate discount factors using the recurrent relation;
4. store them in the cell arrays *dFD* as discount factors for default paths and *dFN* as discount factors for non-default paths respectively;

*Step C* Calculating $\zeta _{kt}$ and $P_{kt}$ for non-default paths.

This step computes forward value of a unit capital invested in *k*th loan with a non-default credit rating migration path *t* and its associated migration probability.

1. for every loan k=1,...,K and for every migration path $t=1,\ldots ,T$, repeat the following;
2. load discount factors obtained by step B (*dFN*);
3. load interest rates deduced from Table 6, Column 3;
4. calculate forward value $\zeta _{kt}$ using Eq. (9);
5. load migration path possibilities obtained from Step A;
6. calculate $P_{kt}$ using Eq. (11) for non-default migration path;
7. compute $\zeta _{kt}\cdot P_{kt}$ for non-default migration path;

*Step D* Calculating $\zeta _{ktd}$ and $P_{kdt}$ for default paths.

This step computes forward value of a unit capital invested in *k*th loan with a default credit rating migration path *dt* and its associated migration probability.

1. for every loan k=1,...,K and for every migration path $t=1,\ldots ,T,$ repeat the following;
2. load discount factors obtained by step B (*dFD*);
3. load recovery rates deduced from Table 2;
4. calculate forward value $\zeta _{kdt}$ using Eq. (9);
5. load migration path possibilities obtained from Step A;
6. calculate $P_{kdt}$ using Eq. (12) for default migration path;
7. compute $\zeta _{kdt}\cdot P_{kdt}$ for default migration path;

*Step E* Estimating $E(\zeta _k\mid \zeta _k \le b)$ and $Var(\zeta _k\mid \zeta _k \le b)$

This step estimates expected forward loan value and variance of unit capital invested in *k*th loan. The steps consist of:

1. for every loan k = 1,...,K, repeat the following;
2. sum all $P_{kt} \cdot \zeta _{kt}$ to get $\mu _k$;
3. compute $\sigma _k^{2}$ using the computed $\mu _k$ as shown in Eq. (14);
4. use the above information to compute expected forward loan value $E(\zeta _k\mid \zeta _k \le b)$ and variance of unit capital $Var(\zeta _k\mid \zeta _k \le b)$ from equations (12) and (13) respectively;

## References

[CR1] Abaffy J, Bertocchi M, Dupačová J, Moriggia V, Consigli G (2007). Pricing nondiversifiable credit risk in the corporate Eurobond market. J Bank Finance.

[CR2] Alizadeh F, Goldfarb D (2003). Second-order cone programming. Math Program.

[CR3] Altman EI (1989). Measuring corporate bond mortality and performance. J Finance.

[CR4] Arnold B, Borio C, Ellis L, Moshirian F (2012). Systemic risk, macroprudential policy frameworks, monitoring financial systems and the evolution of capital adequacy. J Bank Finance.

[CR5] Asarnow E, Edwards D (1995). Measuring loss on defaulted bank loans: a 24-year study. J Commer Lend.

[CR6] Athanasoglou PP, Brissimis SN, Delis MD (2008). Bank-specific, industry-specific and macroeconomic determinants of bank profitability. J Int Financ Mark Inst Money.

[CR7] Barrera J, Homem-de Mello T, Moreno E, Pagnoncelli BK, Canessa G (2014) Chance-constrained problems and rare events: an importance sampling approach, Preprint available at Optimization Online (http://www.optimization-online.org)

[CR8] Benz M (2002) The implications of the “new capital adeqaucy framework” for credit risk and capital management in the banking industry. Diplom.de

[CR9] Berger AN, DeYoung R, Flannery MJ, Lee D, Öztekin O (2008). How do large banking organizations manage their capital ratios?. J Financ Serv Res.

[CR10] Bessis J (2015). Risk management in banking.

[CR11] Bridges J, Gregory D, Nielsen M, Pezzini S, Radia A, Spaltro M (2014) The impact of capital requirements on bank lending. Bank of England Working Paper

[CR12] Brusov P, Filatova T, Orekhova N, Eskindarov M (2015). The global causes of the global financial crisis, modern corporate finance, investments and taxation.

[CR13] Calabrese R, Zenga M (2008). Measuring loan recovery rate: methodology and empirical evidence. Stat Appl.

[CR14] Calabrese R (2014). Predicting bank loan recovery rates with a mixed continuous-discrete model. Appl Stoch Models Bus Ind.

[CR15] Calafiore GC, El Ghaoui L (2006). On distributionally robust chance-constrained linear programs. J Optim Theory Appl.

[CR16] Cannata F, Quagliariello M (2009) The role of Basel II in the subprime financial crisis: guilty or not guilty? CAREFIN Research Paper, 3/09

[CR17] Carty LV, Lieberman D (1996) Corporate bond defaults and default rates 1938–1995. Moodys Investors Service, Global Credit Res

[CR18] Charnes A, Cooper WW (1963). Deterministic equivalents for optimizing and satisficing under chance constraints. Oper Res.

[CR19] Choudhry M (2012). The principles of banking.

[CR20] Crouhy M, Galai D, Mark R (2010) Risk exposures. Encyclopedia of Quantitative Finance, Wiley Online Library

[CR21] Delage E, Mannor S (2010). Percentile optimization for Markov decision processes with parameter uncertainty. Oper Res.

[CR22] Erkens DH, Hung M, Matos P (2012). Corporate governance in the 2007–2008 financial crisis: evidence from financial institutions worldwide. J Corp Finance.

[CR23] Flannery MJ (2014). Maintaining adequate bank capital. J Money Credit Bank.

[CR24] Flannery MJ, Rangan KP (2008). What caused the bank capital build-up of the 1990s?. Rev Finance.

[CR25] Gropp R, Heider F (2010). The determinants of bank capital structure. Rev Finance.

[CR26] Gupton GM, Finger CC, Bhatia M (1997). CreditMetrics: technical document.

[CR27] Hasan I, Siddique A, Sun X (2015). Monitoring the invisible hand of market discipline: capital adequacy revisited. J Bank Finance.

[CR28] Jankowitsch R, Nagler F, Subrahmanyam MG (2014). The determinants of recovery rates in the US corporate bond market. J Financ Econ.

[CR29] Khieu HD, Mullineaux DJ, Yi HC (2012). The determinants of bank loan recovery rates. J Bank Finance.

[CR30] Krug S, Lengnick M, Wohltmann HW (2015). The impact of Basel III on financial (in) stability: an agent-based credit network approach. Quant Finance.

[CR31] Lee E (2014). Basel III and its new capital requirements, as distinguished from basel II. Bank Law J.

[CR32] Luedtke J (2014). A branch-and-cut decomposition algorithm for solving chance-constrained mathematical programs with finite support. Math Program.

[CR33] Maggioni FRANCESCA, Potra FA, Bertocchi MI, Allevi E (2009). Stochastic second-order cone programming in mobile ad hoc networks. J Optim Theory Appl.

[CR34] McNeil AJ, Frey R, Embrechts P (2015). Quantitative risk management: concepts, techniques and tools: concepts, techniques and tools.

[CR35] Prékopa A (1995) Stochastic programming. Math Appl 324

[CR36] Rachev ST (2003). Handbook of heavy tailed distributions in finance: handbooks in finance (vol 1).

[CR37] Rachev ST, Menn C, Fabozzi FJ (2005). Fat-tailed and skewed asset return distributions: implications for risk management, portfolio selection, and option pricing (vol 139).

[CR38] Renault O, Scaillet O (2004). On the way to recovery: a nonparametric bias free estimation of recovery rate densities. J Bank Finance.

[CR39] Saunders A, Allen L (2010). Credit risk management in and out of the financial crisis: new approaches to value at risk and other paradigms.

[CR40] Schuermann Tl (2004) What do we know about loss given default? Working Paper, Federal Reserve Bank of New York. Also in Shimko D (ed) (2006) Credit risk models and management, 2nd edn. Risk Books, London, UK

[CR41] Standard & Poor’s (2014) 2013 Annual global corporate default study and rating transitions. Retrieved from http://www.maalot.co.il/publications/fts20140324161422. pp 53–54

[CR42] Szegö GP (2004). Portfolio theory: with application to bank asset management.

[CR43] Uryasev S (2000). Introduction to the theory of probabilistic functions and percentiles (value-atrisk).

[CR44] Yang W, Xu H (2016). Distributionally robust chance constraints for non-linear uncertainties. Math Program.

[CR45] Zymler S, Kuhn D, Rustem B (2013). Distributionally robust joint chance constraints with second-order moment information. Math Program.

